# Case Report: A Severe Paediatric Presentation of COVID-19 in APDS2 Immunodeficiency

**DOI:** 10.3389/fimmu.2022.881259

**Published:** 2022-05-30

**Authors:** Nuria Sanchez Clemente, Justin Penner, Judith Breuer, Winnie Ip, Claire Booth

**Affiliations:** ^1^ Immunology and Infectious Diseases Department, Great Ormond Street Hospital for Children, London, United Kingdom; ^2^ Department of Infectious Disease Epidemiology, London School of Hygiene & Tropical Medicine, London, United Kingdom; ^3^ Infection, Immunity & Inflammation Department, UCL Great Ormond Street Institute of Child Health, London, United Kingdom; ^4^ Department of Paediatric Immunology, Great Ormond Street Hospital for Children NHS Trust, London, United Kingdom

**Keywords:** primary immunodeficiency, APDS2, SARS-CoV-2, COVID-19, case report

## Abstract

Critical respiratory manifestations of severe acute respiratory syndrome coronavirus-2 (SARS-CoV-2) are rare in children, and little is known about how immunocompromised children respond to the infection. We report a case of a 4-year-old boy with activated PI3K delta syndrome type 2 (APDS2) with a protracted and severe COVID-19 course with both inflammatory and acute respiratory features. He was treated with remdesivir, nitazoxanide, high-dose corticosteroids, and tocilizumab and made a full recovery. We propose that remdesivir may be used in combination with nitazoxanide to improve viral clearance and reduce the chance of resistance in treating acute SARS-CoV-2 infection.

## Introduction

Severe acute respiratory syndrome coronavirus-2 (SARS-CoV-2) infection in children rarely results in critical respiratory illness. A small proportion of children present with a severe post-infectious inflammatory condition known as paediatric inflammatory multisystem syndrome temporally associated with SARS-CoV-2 (PIMS-TS), and an even smaller proportion experience a combination or sequential manifestations of coronavirus disease 2019 (COVID-19). It is still unclear whether immunocompromised children are at higher risk of complications secondary to either of these clinical entities (1–4), although children with co-morbidities involving more than one body system do seem to be at higher risk of severe disease and death (1). A number of individual case reports describe paediatric patients with primary immunodeficiency (PID) experiencing asymptomatic infection or mild illness followed by SARS-CoV-2 clearance (5, 6). Increased morbidity and mortality from COVID-19 have been described in adult PID patients (7), particularly those with combined immune deficiency and immune dysregulation (8), as well as protracted disease courses in antibody-deficient patients (9).

## Case Description

We describe a case of a 4-year-old boy with activated PI3K delta syndrome type 2 (APDS2) with a protracted COVID-19 course and both inflammatory and acute respiratory features that resulted in admission to our paediatric intensive care unit (PICU). APDS2 is a PID characterised by recurrent respiratory infections (bronchiectasis is a common complication), persistent or recurrent herpes virus infections, lymphadenopathy, splenomegaly, increased risk of lymphoma, and occasionally, developmental delay and growth retardation (10).

The patient is the second child of non-consanguineous Vietnamese parents born at term. He had a normal neonatal period until the age of 1 when he developed recurrent chest infections requiring multiple courses of antibiotics. He also had several episodes of wheezing requiring nebuliser treatment and hospital admission.

During one presentation of wheezing at age 2 years 6 months, he was found to have splenomegaly. Abdominal ultrasound and CT chest demonstrated widespread lymphadenopathy. Lymph node biopsy showed detectable adenovirus and Epstein–Barr virus (EBV) and histology consistent with reactive changes and no evidence of malignancy. Bone marrow aspirate and trephine revealed hypercellular marrow, adequate trilineage haematopoiesis, and no evidence of malignancy.

### Diagnostic Assessment

Immunological investigations at this time showed normal functional apoptosis, vitamin B12 level, CD3/28 stimulation response, CTLA4 recycling pathway, and normal TH17 cell profiling, making ALPS and CTLA4 haploinsufficiency unlikely diagnoses. The patient did however have an increased double-negative T-cell population (3.9%–5%), raised sFASL at 461 (normal < 200 pg/ml), low naive T cells (34% naive CD4 and CD8), increased memory CD8 proportion (50%), and low CD4+ and CD8+ TREC levels (see **Table 1** in **Appendix 1**). Immunoglobulins showed a dysregulated profile with raised IgG 21.2–24 g/L and IgM 3.74–4.8 g/L; IgA was normal. Vaccine responses to pneumococcal antigens were poor. Targeted panel genetics identified a heterozygous mutation in PIK3R1 (NM_181523.2:c.243A>T p.(Lys81Asn), not present in the gnomAD database. Heterozygous mutations in this gene have been described to cause APDS2.

The patient responded well to sirolimus resulting in complete regression of hepatosplenomegaly and significant improvement of lymphadenopathy. Antibiotic prophylaxis with azithromycin and immunoglobulin replacement was commenced. He received rituximab therapy for ongoing EBV viraemia leading to clearance of the virus and depletion of B cells.

Haematopoietic stem cell transplantation (HSCT) from a matched sibling donor was planned as a curative therapeutic option, and he was admitted at the age of 4 years 6 months in preparation for this. At this time, he had a 2-week history of wet cough. His father had similar symptoms and was PCR positive for SARS-CoV-2 on a respiratory swab, as did his sibling donor, although the patient remained negative. He was not eligible for COVID vaccination at this time. HSCT was postponed.

One week later, he presented to his local hospital with a fever of 40°C and abdominal pain. He was initially treated with empiric piperacillin/tazobactam and vancomycin cover for central line sepsis. Due to persistent fever, antibiotics were changed to meropenem and vancomycin. He experienced a degree of acute renal impairment (creatinine elevated to 135 μmol/L) and received intravenous (IV) fluids. A differential diagnosis of COVID-19 infection was considered in light of family members testing positive and suggestive chest X-ray (CXR) changes; however, his nasopharyngeal aspirate (NPA) was initially negative for SARS-CoV-2 by reverse transcriptase–PCR (rtPCR).

He was transferred to our hospital after 6 days, with an equivocal SARS-CoV-2 PCR NPA result on admission followed by two negative NPA results 24 and 72 h later. However, his stool was found to be rtPCR positive (cT 30 and 27) on days 2 and 8 of admission. He remained febrile and was noted to be lymphopaenic (0.51 × 10^9^/L) and thrombocytopaenic (42 × 10^9^/L) with a raised lactate dehydrogenase (LDH) (4,798 U/L) and increasingly inflammatory blood biomarkers with an erythrocyte sedimentation rate (ESR) >170 and ferritin of 2,145 μg/L.

**Figure 1:**
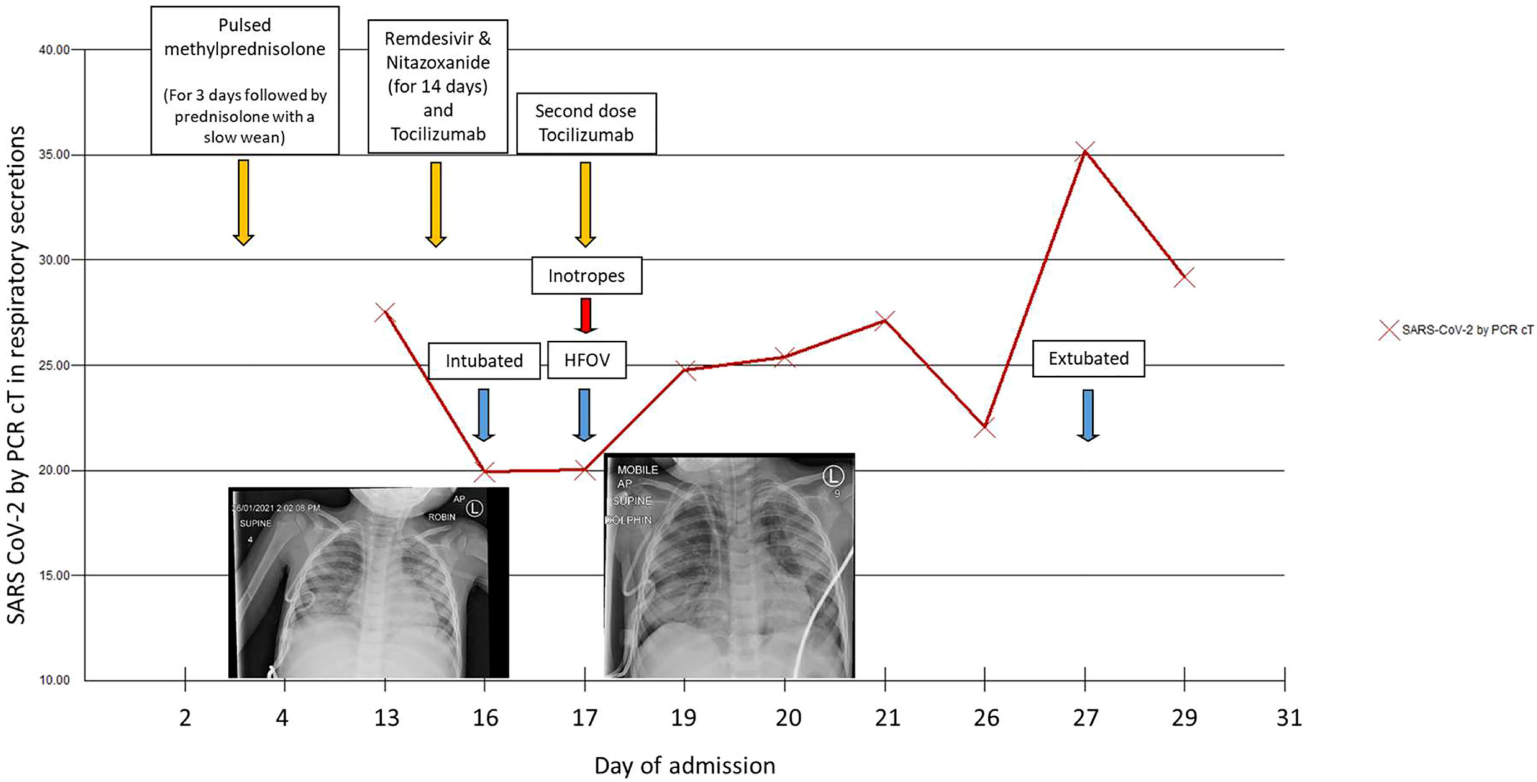
SARS CoV-2 cycle threshold (cT) over time and chest X-rays from Day 13 (left) showing widespread patchy airspace opacification and Day 16 (right) post-intubation showing a large volume pneumomediastinum, with “continuous diaphragm” sign and gas extending superiorly into the neck and inferiorly below the diaphragm.

Following a multidisciplinary team meeting on day 3 of admission, the decision was made to treat with pulsed methylprednisolone 10 mg/kg for 3 days while continuing broad-spectrum antibiotics to treat for possible PIMS-TS or a haemophagocytic lymphohistiocytosis (HLH) picture (see **Table 2** in **Appendix 1** for a full panel of results). Fevers settled, and he made a clinical improvement. However, he developed an oxygen requirement on day 13 with progressively increased work of breathing. CXR showed widespread patchy airspace opacification in keeping with COVID-19 pneumonitis (see **Figure 1**). NPAs subsequently came back positive for SARS-CoV-2 (cT 27), which on genotyping was found to be the alpha variant.

### Therapeutic Intervention

Non-invasive ventilation with Optiflow was started, but there was a rapid deterioration requiring transfer to PICU for respiratory support on day 14. He was started on compassionate-use remdesivir (5 mg/kg loading dose followed by 2.5 mg/kg daily) and nitazoxanide (500 mg BD), based on *in vitro* evidence of its inhibition of SARS-CoV-2 at low-micromolar concentration (11) as well as tocilizumab (200 mg) for potential anti-inflammatory effects in COVID-19. He quickly required escalation to conventional ventilation, and on day 16, he developed a pneumomediastinum and was transitioned to high-frequency oscillatory ventilation (HFOV). Inotropic support was briefly required; echocardiography at this point was unremarkable. Ventilation remained challenging, and he required intermittent proning to maintain oxygenation. He improved slowly and was extubated to Optiflow after 11 days of invasive ventilation.

He received a total of 14 days of remdesivir and nitazoxanide with no signs of toxicity. As well as the antibacterial cover, he received empiric antifungal therapy with liposomal amphotericin B and later posaconazole. He was continued on 2 mg/kg oral prednisolone with a slow wean following pulsed methylprednisolone. With deterioration and progression to HFOV, his antibiotics were escalated to meropenem, and one further dose of tocilizumab was given.

From day 25, the patient had intermittent fevers with no clear source of infection and no rise in inflammatory markers. This was attributed to possible drug fever from piperacillin/tazobactam, with a resolution of fever episodes upon cessation. SARS-CoV-2 viral load gradually reduced in his respiratory secretions as evidenced by an increase in cT value from 20 to 35 over 2 weeks and viral clearance in stool and NPA after 4 weeks. His COVID serology was checked several times and remained negative.

Following 15 days of intensive care with a significant period of neuromuscular blockade, he developed a critical care myopathy requiring significant rehabilitation. The patient was discharged home after a 6-week stay with his general condition not yet returned to baseline. HSCT was delayed for 3 months to allow for further recovery.

## Discussion

In summary, we report the first case of severe COVID-19 pneumonitis with a significant inflammatory component in a paediatric patient with APDS2 immunodeficiency who was successfully treated with remdesivir, nitazoxanide, high-dose corticosteroids, and tocilizumab. To our knowledge, this is the first report of life-threatening COVID-19 infection in the context of APDS2 immunodeficiency where previous descriptions of the infection in adults with APDS in an international inborn error of immunity cohort described a mild course (12).

This report provides *in vivo* evidence to support the use of remdesivir in combination with nitazoxanide to improve viral clearance and reduce the chance of resistance in treating acute SARS-CoV-2 (13). The greater transmissibility of the new Omicron variant has led to increased SARS-CoV-2 infections in children including those with underlying PID. Optimal treatment in this population remains uncertain, and patients are typically managed on a case-by-case basis in multidisciplinary teams, as occurred with our patient. The re-purposing of medications with potential SARS-CoV-2 antiviral properties, such as nitazoxanide and fluvoxamine in combination with remdesivir, may be particularly beneficial in this population in whom prolonged viraemia has been demonstrated. New, oral preventative antivirals with activity against SARS-CoV-2 such as the RNA-dependent RNA polymerase (RdRp) inhibitors molnupiravir and favipiravir, the latter of which have previously proved effective in combination regimens (14) for the treatment of other RNA viruses such as influenza B, as well as nirmatrelvir and lopinavir/ritonavir (15), are also emerging treatment options for mild disease in the PID population who are at risk for progression to severe disease. However, evidence of their use in children is currently lacking, and drug-to-drug interactions may be particularly problematic in this patient population who are often on several medications already. Similarly, the use of monoclonal antibodies, i.e., sotrovimab or Regeneron (casirivimab and imdevimab) (16) either alone or in combination with antivirals such as remdesivir may prove beneficial for patients such as ours with limited B-cell response to infection and/or who have been treated with rituximab to prevent severe progression to both COVID-19 pneumonia and inflammatory complications of SARS-CoV-2. Ultimately, the risk of SARS-CoV-2 disease in children with PID and the design of optimal treatment pathways for this population, both in the acute phase and for the prevention of progression to a hyperinflammatory state, require further study.

### Patient Perspective

We received written informed consent from the patient’s parents for the publication of this case. The patient received a matched sibling donor HSCT following recovery from this episode and is now 8 months post-transplant with mixed donor chimerism and good immune reconstitution. He became SARS-CoV-2 positive 7 months post-transplant, remained asymptomatic, and cleared the virus without any additional treatment.

## Data Availability Statement

The original contributions presented in the study are included in the article/**Supplementary Material**. Further inquiries can be directed to the corresponding author.

## Ethics Statement

Written informed consent was obtained from the minor(s)’ legal guardian/next of kin for the publication of any potentially identifiable images or data included in this article.

## Author Contributions

NSC and CB wrote the manuscript. CB, WI, and JP treated the patient and, along with JB, provided treatment recommendations. NSC and JP collected the clinical data. All authors critically reviewed the manuscript.

## Funding

Wellcome Trust funded the work of JB, and all research at GOSH is supported by the NIHR BRC.

## Conflict of Interest

The authors declare that the research was conducted in the absence of any commercial or financial relationships that could be construed as a potential conflict of interest.

## Publisher’s Note

All claims expressed in this article are solely those of the authors and do not necessarily represent those of their affiliated organizations, or those of the publisher, the editors and the reviewers. Any product that may be evaluated in this article, or claim that may be made by its manufacturer, is not guaranteed or endorsed by the publisher.
