# Supplementary material for: Case Report: A Severe Paediatric Presentation of COVID-19 in APDS2 Immunodeficiency
Source: Front Immunol. 2022 May 30;13:881259. doi: 10.3389/fimmu.2022.881259 (PMC9190774; doi:10.3389/fimmu.2022.881259)
Supplement: Supplementary file 1 [file DataSheet_1.docx]

Appendix 1.

Table 1. Laboratory investigations showing HLH markers during the patient’s acute illness.

|  | At deterioration | Peak value | Normal range |
| --- | --- | --- | --- |
| Hb (g/l) | 6.3 | 9.5* | 11.5-14.5 |
| WCC (x10*9/l) | 1.02 | 0.36 | 5-15 |
| Neutrophils | 0.36 | 0.08 | 1.5-8.5 |
| Lymphocytes | 0.56 | 0.27 | 2-9.5 |
| Platelets | 50 | 44 | 150-450 |
|  |  |  |  |
| Ferritin (ug/l) | 992 | 9985 | 13.4-75 |
| Triglycerides (mmol/l) | 5.18 | 27.25 | 0.36-1.3 |
|  |  |  |  |
| ALT (u/l) | 68 | 133 | 10-25 |
| GGT (u/l) | 19 | 22 | 10-22 |
| Bilirubin (umol/l) | 8 | 16 | <18 |
|  |  |  |  |
|  |  |  |  |

*transfused

Table 2. Immunological diagnostic investigations done during the work-up of the patient’s primary immune deficiency

|  | Patient | Control | Ref range |
| --- | --- | --- | --- |
|  |  |  |  |
| CD3+ (x10*9/l) | 1.73 |  |  |
| CD3% | 43 |  |  |
| CD19+ | 1.89 |  |  |
| CD19% | 47 |  |  |
| CD16/56+ | 0.38 |  |  |
| CD16/56% | 9 |  |  |
| CD3+4+ | 0.94 |  |  |
| CD4% | 23 |  |  |
| CD3+8+ | 0.58 |  |  |
| CD8% | 15 |  |  |
|  |  |  |  |
| Naïve CD4+ (CD4+RA+27+) | 0.32 |  |  |
| Naïve CD4% | 34 |  |  |
| Memory CD4+ (CD4+RA-27+) | 0.59 |  |  |
| Memory CD4% | 63 |  |  |
| Effector CD4+ (CD4+RA+27-) | 0 |  |  |
| Effector CD4% | 0 |  |  |
| Naïve CD8+ (CD8+RA+27+) | 0.23 |  |  |
| Naïve CD8% | 39 |  |  |
| Memory CD8+ (CD8+RA-27+) | 0.29 |  |  |
| Memory CD8% | 50 |  |  |
| Effector CD8+ (CD8+RA+27-) | 0.01 |  |  |
| Effector CD8% | 1.6 |  |  |
|  |  |  |  |
| Gamma delta cells | 0.12 |  |  |
| Gamma delta cell % | 6.7 |  |  |
| CD3+4-8- | 0.06 |  |  |
| CD3+4-8- % | 3.8 |  | No age spec ref range |
|  |  |  |  |
| sFasL (pg/ml) | 461 |  | <200pg/ml |
| Serum B12 (pg/ml) | 834 |  | 264-1220 |
| Functional apoptosis |  |  |  |
| *Annexin V+ cells %* | 32 | 26 |  |
| *Fold change in death* | 16.1 | 17.3 | >2 fold change |
|  |  |  |  |
| PHA stimulation | Normal |  |  |
| CD3/28 stimulation | Normal |  |  |
|  |  |  |  |
| TH17 assay |  |  |  |
| *CD4+%* | 26 | 60 |  |
| *CD4+RO+ %* | 60 | 35 |  |
| *CD4+RO- %* | 40 | 64 |  |
| *CD4+RO+IL4+ %* | 1.6 | 0.9 |  |
| *CD4+RO+IFNg %* | 1.3 | 12.5 |  |
| *CD4+IL17+ %* | 0.9 | 2.2 | Research assay, TH17 >0.04% normal |
|  |  |  |  |
| CTLA4 upregulation on memory FoxP3 cells | Normal |  |  |
|  |  |  |  |
| STAT3 phosphorylation to IL-6 stimulation (%) | 24 | 59 |  |
| STAT3 phosphorylation MFI | 9 | 19 |  |
|  |  |  |  |
| LDH (u/l) | 465 |  | 500-920 |
| Ferritin (ng/ml) | 22 |  | 4.2-62 |
|  |  |  |  |
| IgG (g/l) | 24.1 |  | 4.9-16 |
| IgA | 0.98 |  | 0.4-2 |
| IgM | 4.8 |  | 0.5-2 |
|  |  |  |  |
| ESR (mm/hr) | 70 |  | 0-10 |
|  |  |  |  |
| SAP/XIAP expression | Normal |  |  |
|  |  |  |  |
| Bone marrow analysis |  |  |  |
| Morphology | Normal |  |  |
| FISH analysis | Normal |  |  |
| Cytogenetic analysis | Normal |  |  |
| Lymphoid Immunophenotyping | Normal |  |  |
|  |  |  |  |
